# Dasatinib May Override F317L BCR-ABL Kinase Domain Mutation in Patients with Chronic Myeloid Leukemia

**DOI:** 10.4274/Tjh.2012.0013

**Published:** 2013-06-05

**Authors:** Ahmet Emre Eşkazan, Teoman Soysal

**Affiliations:** 1 Diyarbakır Training and Research Hospital, Department of Hematology, Diyarbakır, Turkey; 2 İstanbul University Cerrahpaşa Faculty of Medicine, Department of Internal Medicine, Division of Hematology, İstanbul, Turkey

## TO THE EDITOR

The most common mechanisms for resistance in patients with chronic myeloid leukemia (CML) receiving imatinib mesylate are BCR-ABL kinase domain (KD) mutations. The mutations are stratified according to in vitro 50% inhibitory concentration (IC50) values. The F317L KD mutation has been shown to induce a 9- to 13.5-fold increase of dasatinib IC50 with respect to wild-type BCR-ABL in cellular assays [1]. The pharmacokinetic data showed that F317L is predicted to be moderately sensitive to dasatinib [2]. Jabbour et al. [3] published data on a cohort of 20 CML patients with F317L mutation and evaluated the characteristics and outcomes of these patients with tyrosine-kinase inhibitor (TKI) therapy. Among these 20 patients, F317L was detected in 12 after imatinib failure and in 8 after dasatinib failure. In the post-imatinib failure group, 3 patients received dasatinib. The best achieved response was partial hematologic response in 1 and complete hematologic response (CHR) in 2. Müller et al. [4] also reported the results of analysis of original dasatinib phase 2/3 trial data according to pre-existing mutations. Of the 402 patients with baseline KD mutations, 14 had F317L mutations; 13 (93%), 2 (14%), and 1 (7%) achieved CHR, major cytogenetic response (MCyR), and complete cytogenetic response (CCyR), respectively. None of the patients achieved major molecular response (MMR). 

Among our CML cohort, we identified KD mutations by the denaturing high performance liquid chromatography sequencing method as described before [5]. In the literature, it has been recently demonstrated by our group as well as by some others that there are CML patients with F317L mutation who achieved and maintained both CCyR and MMR with dasatinib after imatinib failure (Table 1) [6,7,8,9]. 

Dasatinib is known to have significant therapeutic activity against the Src kinases, and this is responsible for several of its “off-target effects”. Pulmonary toxicity [i.e. pleural effusion (PE)] following dasatinib use can be observed in CML patients [10]. Lymphocytosis in CML patients receiving dasatinib might have contributed to therapeutic efficacy, and Mustjoki et al. [11] showed a strong association between clonal T/NK cell expansion and lymphocytic PE under dasatinib therapy and prolonged stable responses in patients with advanced Ph-positive leukemias. Among the 4 CML cases described in the literature with F317L in which MMR was achieved with dasatinib, 3 had episodes of reversible dasatinib-induced PEs and modest lymphocytosis was seen in 1 (Table 1). These clinical and laboratory findings may be attributed to the good response in these patients. 

In conclusion, evidence indicating the resistance of the F317L mutation to TKIs mainly comes from in vitro studies. Since mutations are classified on the basis of their in vitro sensitivity to TKIs, the in vivo outcomes may not be in accordance with in vitro studies in every case. The presence of the F317L KD mutation may not uniformly predict poor outcomes in CML patients mainly on dasatinib. Outcomes may be related to a complex interplay of several factors possibly including the off-target effects of the TKI. It is important to carefully investigate and monitor individual patients, and although a switch to a TKI with better in vitro potency against a mutation may improve outcome, this strategy might not always be necessary, especially if the patient is in durable cytogenetic or molecular response. 

## CONFLICT OF INTEREST

None of authors of this paper has any conflicts of interest, including specific financial interests, relationships, and/or affiliations, relevant to the subject matter or materials included in this manuscript.

## Figures and Tables

**Table 1:**
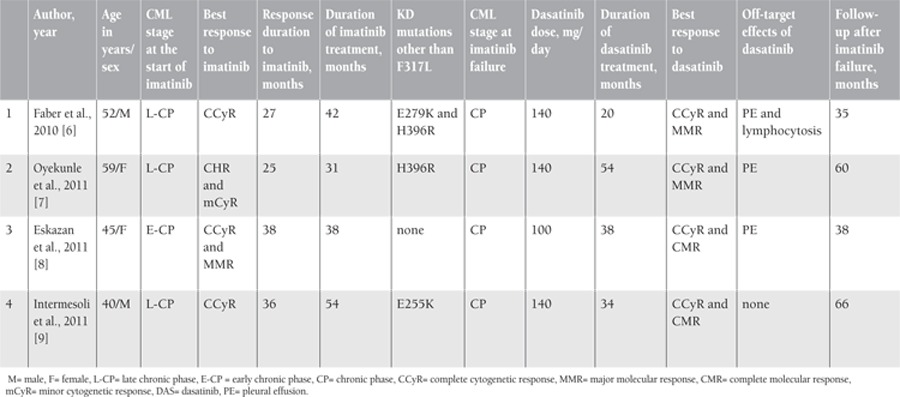
Characteristics of CML patients with F317L mutation with MMR under dasatinib published in the literature.

## References

[1] O’Hare T, Walters DK, Stoffregen EP, Jia T, Manley PW, Mestan J, Cowan-Jacob SW, Lee FY, Heinrich MC, Deininger MW, Druker BJ (2005). In vitro activity of Bcr-Abl inhibitors AMN107 and BMS-354825 against clinically relevant imatinib-resistant Abl kinase domain mutants. Cancer Res.

[2] Laneuville P, Dilea C, Yin OQ, Woodman RC, Mestan J, Manley PW (2010). Comparative In vitro cellular data alone are insufficient to predict clinical responses and guide the choice of BCR-ABL inhibitor for treating imatinib-resistant chronic myeloid leukemia. J Clin Oncol.

[3] Jabbour E, Kantarjian HM, Jones D, Reddy N, O’Brien S, Garcia-Manero G, Burger J, Cortes J (2008). Characteristics and outcome of chronic myeloid leukemia patients with F317L BCR-ABL kinase domain mutation after therapy with tyrosine kinase inhibitors. Blood.

[4] Müller MC, Cortes JE, Kim DW, Druker BJ, Erben P, Pasquini R, Branford S, Hughes TP, Radich JP, Ploughman L, Mukhopadhyay J, Hochhaus A (2009). Dasatinib treatment of chronic-phase chronic myeloid leukemia: analysis of responses according to preexisting BCR-ABL mutations. Blood.

[5] Erbilgin Y, Çatal S, Eşkazan AE, Hatırnaz Ö, Sosyal T, Özbek U (2011). ABL gene kinase domain mutation scanning by denaturing high performance liquid chromatography sequencing method. Turk J Hematol.

[6] Faber E, Mojzikova R, Plachy R, Rozmanova S, Stastny M, Divoka M, Jarosova M, Indrak K, Divoky V (2010). Major molecular response achieved with dasatinib in a CML patient with F317L BCR-ABL kinase domain mutation. Leuk Res.

[7] Oyekunle AA, Castagnetti F, Gugliotta G, Soverini S, Baccarani M, Rosti G (2011). F317L BCR-ABL1 kinase domain mutation associated with a sustained major molecular response in a CML patient on dasatinib. Leuk Res.

[8] Eskazan AE, Soysal T, Erbilgin Y, Ozbek U, Ferhanoglu B (2011). Chronic myeloid leukemia patients with F317L BCR-ABL kinase domain mutation are resistant to dasatinib: is that true for all the patients?. Leuk Res.

[9] Intermesoli T, Castagnetti F, Soverini S, Bussini A, Spinelli O, Gnani A, Bassan R, Rosti G (2012). Durable molecular response despite F317L and E255K mutations: Successful treatment of chronic myeloid leukemia with sequential imatinib, nilotinib and dasatinib. Leuk Res.

[10] Eskazan AE, Soysal T, Ongoren S, Gulturk E, Ferhanoglu B, Aydin Y (2011). Pleural and pericardial effusions in chronic myeloid leukemia patients receiving low-dose dasatinib therapy. Haematologica.

[11] Mustjoki S, Ekblom M, Arstila TP, Dybedal I, Epling-Burnette PK, Guilhot F, Hjorth-Hansen H, Höglund M, Kovanen P, Laurinolli T, Liesveld J, Paquette R, Pinilla-Ibarz J, Rauhala A, Shah N, Simonsson B, Sinisalo M, Steegmann JL, Stenke L, Porkka K (2009). Clonal expansion of T/NK-cells during tyrosine kinase inhibitor dasatinib therapy. Leukemia.

